# Retraction: Physical exercise and psychological health of rural left-behind children: an experiment from China

**DOI:** 10.3389/fpsyg.2023.1256019

**Published:** 2023-07-14

**Authors:** Frontiers Editorial Office

Following publication, the publisher uncovered evidence that a false identity was used in the peer-review process. The assignment of the false identity was confirmed by an investigation conducted in accordance with Frontiers' policies and the Committee on Publication Ethics (COPE) guidelines.

Because the peer-review had been compromised, the investigation included a post-publication review of the article, which concluded that the article should have been rejected because it does not meet the standards for publication in Frontiers in Psychology.

This retraction was approved by the Chief Editors of Frontiers in Psychology and the Chief Executive Editor of Frontiers. The authors do not agree to this retraction.

